# Case report: JAK inhibition as promising treatment option of fatal RVCLS due to *TREX1* mutation (pVAL235Glyfs^*^6)

**DOI:** 10.3389/fneur.2023.1118369

**Published:** 2023-02-21

**Authors:** Friederike Ufer, Susanne M. Ziegler, Marcus Altfeld, Manuel A. Friese

**Affiliations:** ^1^Institute of Neuroimmunology and Multiple Sclerosis, University Medical Center Hamburg-Eppendorf, Hamburg, Germany; ^2^Department of Virus Immunology, Leibniz Institute for Virology, Hamburg, Germany

**Keywords:** *TREX1*, brain vascular disorder, hereditary autoinflammatory diseases, *CXCL10*, immunosuppression, JAK inhibition

## Abstract

**Introduction:**

Autosomal dominant mutations in the C-terminal part of *TREX1* (pVAL235Glyfs^*^6) result in fatal retinal vasculopathy with cerebral leukoencephalopathy and systemic manifestations (RVCLS) without any treatment options. Here, we report on a treatment of a RVCLS patient with anti-retroviral drugs and the janus kinase (JAK) inhibitor ruxolitinib.

**Methods:**

We collected clinical data of an extended family with RVCLS (*TREX1* pVAL235Glyfs^*^6). Within this family we identified a 45-year-old woman as index patient that we treated experimentally for 5 years and prospectively collected clinical, laboratory and imaging data.

**Results:**

We report clinical details from 29 family members with 17 of them showing RVCLS symptoms. Treatment of the index patient with ruxolitinib for >4 years was well-tolerated and clinically stabilized RVCLS activity. Moreover, we noticed normalization of initially elevated *CXCL10* mRNA in peripheral blood monocular cells (PBMCs) and a reduction of antinuclear autoantibodies.

**Discussion:**

We provide evidence that JAK inhibition as RVCLS treatment appears safe and could slow clinical worsening in symptomatic adults. These results encourage further use of JAK inhibitors in affected individuals together with monitoring of *CXCL10* transcripts in PBMCs as useful biomarker of disease activity.

## 1. Introduction

Mutations in the C-terminal coding sequence of *TREX1* lead to a defective intracellular localization of TREX1 and dysregulation of oligosaccharyltransferase (OST) activity without affecting DNase activity (1). Clinically, these mutations result in adult-onset retinal vasculopathy with cerebral leukoencephalopathy and systemic manifestations (RVCLS) (2). No treatment options exist and patients usually die within 10 years of symptom onset due to progressive multi-organ damage. However, currently there is no clear evidence that RVCL-related *TREX1* mutations are associated with a primary disturbance of immunological functions. Here, we describe a Caucasian family with RVCLS and report an encouraging treatment response in one of the family members. Our observations might help treating other affected families since approximately 40% of all kindreds reported for RVCLS carry the same pVAL235Glyfs^*^6 *TREX1* mutation.

## 2. Case description

We reviewed the clinical course, laboratory and neuroradiological findings of a Caucasian family affected with RVCLS due to frameshift mutation in *TREX1* (pVAL235Glyfs^*^6). Medical information was obtained by clinical examination and diagnostics at our outpatient clinic, gathered by medical records or by telephone calls with patients and/or treating physicians.

**Figure 1:**
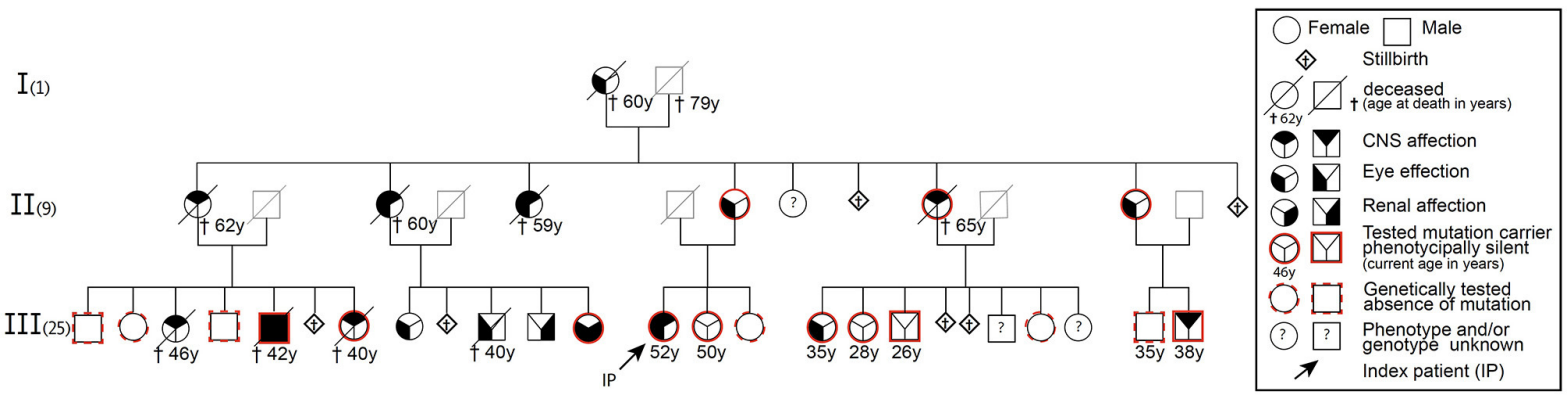
Pedigree of a family with autosomal dominant mutation in the C-terminal sequence of the *TREX1* gene (pVAL235Glyfs^*^6) leading to RVCLS.

We identified 17 individuals with RVCLS symptoms, including eight genetically confirmed mutation carriers (Figure 1). Genetic testing in nine symptomatic individuals was not possible, as they had already died. Symptomatic individuals showed frequent (58%) cerebral abnormalities and retinopathy, while severe kidney disease was less frequent (17%; Supplementary Table S1). In addition, most symptomatic individuals reported systemic symptoms such as migraine, Raynaud syndrome, hepatological abnormalities, fatigue, and arthralgia.

In February 2015 we identified a symptomatic 45-year-old female mutation carrier in this family with a failed treatment response to various immunosuppressive drugs before her RVCLS diagnosis was confirmed. Under the hypothesis—later turned out to be incorrect—of a pathological accumulation of intracellular retroviral elements due to an impaired function of the exonuclease activity of TREX1 (3), we treated this index patient with a combination of anti-retroviral drugs (emtricitabine, tenofovir, nevirapine) (Figure 1, Supplementary material case synopsis and Supplementary Table S2). At various timepoints we collected blood samples. We assumed a similar impaired function of TREX1 as described in the context of other type I interferonopathies like Aicardi-Goutières syndrome or familial chilblain lupus (2). Therefore we isolated RNA from snap-frozen pellets of 5 – 10 × 10^6^ PBMCs and performed rtPCR for common interferon-stimulated genes (*MX1, MX2, ISG15, IFI44L, IFL27, OAS1-3, IFL6, IFL44, TAP1, IFITM3, INF*α*2, INFb2, IFIT3, LY6E*) from our index patient and controls that were matched for age, sex and time of sampling. However, we did not detect any transcriptional interferon response in the PBMCs of our index patient, which challenged our initial hypothesis that the pVAL235Glyfs^*^6 *TREX1* mutation results in a type I interferonopathy. Progressive clinical worsening and further MRI lesion accumulation finally led us to terminate anti-retroviral therapy in January 2016.

**Figure 2:**
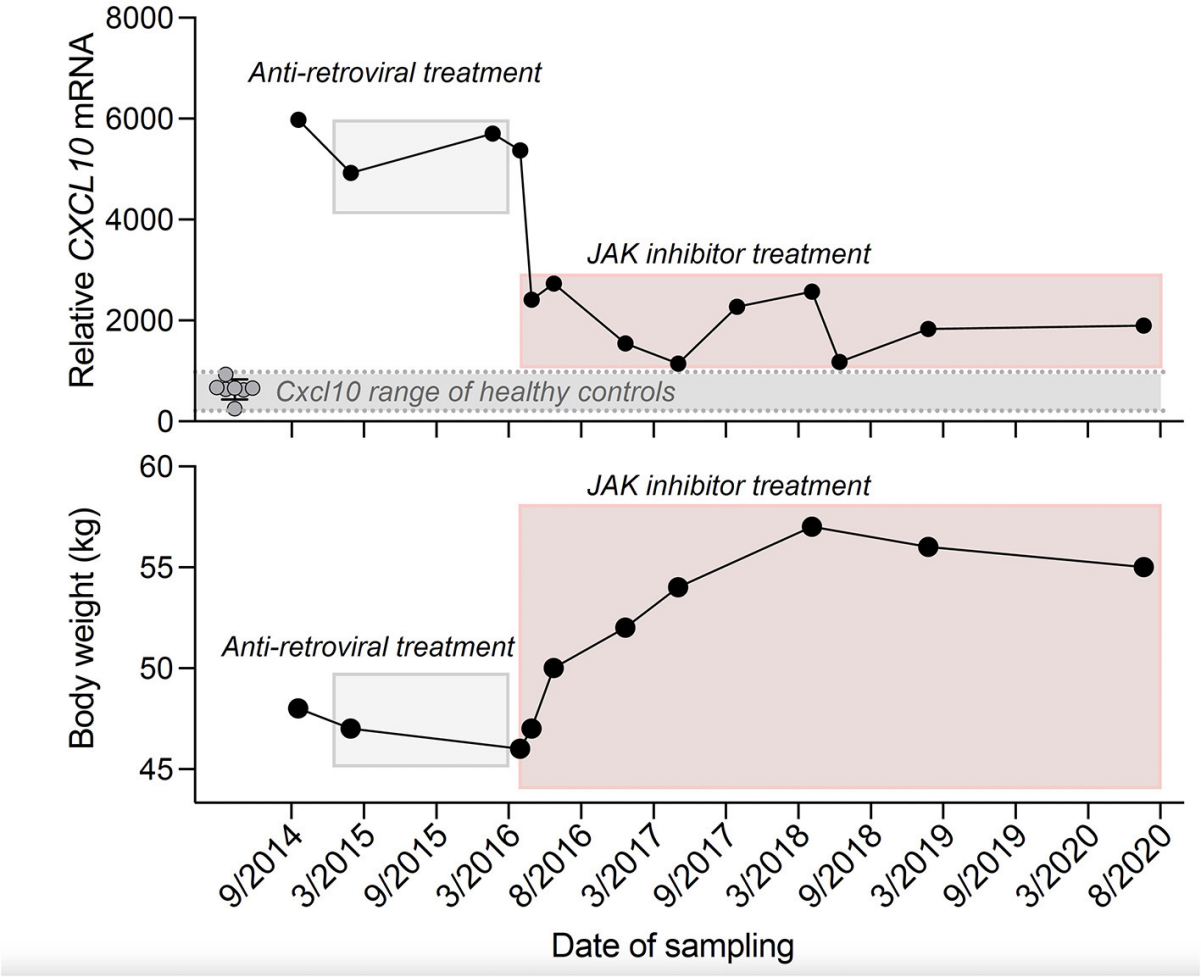
Time course of *CXCL10* mRNA transcript expression in PBMCs of the index patient and eight age- and sex-matched healthy controls **(Top)** and body weight of the index patient **(Bottom)**. RT-PCR results were normalized to TATA-binding-protein **(Top)**. Anti-retroviral treatment: Emtricitabine (200 mg per day), Tenofovir disoproxil fumarate (245 mg per day), Nevirapine (400 mg per day) and JAK inhibitor treatment: Ruxolitinib (10–25 mg per day). *In 09/2014 the patient was not taking any immunosuppressive drugs.

Notably, at that time it was reported that OST dysregulation in pVAL235Glyfs^*^6 *TREX1* mutation carriers was independent of exonuclease function (1) and resulted in release of free glycans that induce autoantibody production (4). Moreover, in human lymphoblasts from RVCL patients caused by mutations in the C-terminal part of *TREX1* an elevated *CXCL10* mRNA has been described (1). Indeed, in the index patient we observed elevated antinuclear autoantibodies (ANA), together with elevated *CXCL10* transcripts in PBMCs at several time points (Supplementary Table S3, Figure 2).

Therefore, in March 2016 we started a treatment with the janus kinase (JAK) inhibitor ruxolitinib that had previously been shown to lower CXCL10 concentrations (5). Of note, clinical disease activity, as measured by newly occurring visual or neurological deficits, stabilized under ruxolitinib. In repeated cMRI scans, we found T2/FLAIR hyperintense white matter lesions in different brain regions (Supplementary Figures S1A, B) and contrast-enhancing lesions particularly in the cerebellum (Supplementary Figure S1C). This lesion burden showed a slower increase under ruxolitinib treatment. Further deterioration of visual acuity of the index patient was stopped and even improved in the left eye from 0.05 to 0.2 and also microbleeds were reduced. In parallel, we found a persistent drop in *CXCL10* transcripts, ANA titers decreased and body weight stabilized (Figure 2).

Ruxolitinib was overall well tolerated. Since anemia and lymphopenia persisted, we reduced ruxolitinib from 25 mg to 10 mg daily while the patient remained clinically stable. However, as anemia and lymphopenia only partially responded to ruxolitinib dose reduction, we also partly assigned it to the disease itself.

## 3. Discussion

Our observations of heterogenous clinical symptoms of RVCLS patients are in line with other reported families (6). In an attempt to ameliorate the clinical disease course in a hypothesis-driven N-of-1 trial we treated the index patient sequentially with reverse-transcriptase (RT) and JAK inhibitors. While RT inhibition showed no effect, JAK inhibition resulted in a marked clinical stabilization.

While accumulations of retroviral elements have been reported in patients and animals with *TREX1* mutations (7), they do not seem to be accumulating in patients with the pVAL235Glyfs^*^6 frameshift mutation in *TREX1* that does not affect the DNase activity. This could explain the ineffectiveness of our RT inhibitor treatment. By contrast, the effectiveness of JAK inhibitors might be explained by their broad anti-inflammatory potential on diverse cytokine pathways including suppression of CXCL10 release (8). CXCL10 has been shown to modulate angiogenesis (9) and was proposed as a major contributor to the vasculopathy observed in RVCLS. There is evidence that CXCL10 inhibits angiogenesis through its receptor CXCR3 which is primarily expressed on activated lymphocytes but also on epithelial and endothelial cells (9). CXCL10 is also induced by the NFκB pathway, but further studies are warranted that investigate this connection to RVCLS. Since ruxolitinib is a non-selective JAK inhibitor it modulates diverse cytokine signaling pathways, hence careful monitoring is needed to evaluate its long-term effects. However, as other interferon-stimulated genes were not elevated in the index patient, we are reluctant to regard pVAL235Glyfs^*^6 frameshift mutation in *TREX1* as an interferonopathy.

Of note, throughout this long period of treatment—including a treatment failure in the beginning—the patient was very motivated, compliant and thankful. She encouraged us, to make this experimental therapy available to other family members. The stabilization of body weight and the regain and preservation of at least limited vision were perceived as most important to her. Body weight was a major health concern of the patient and the ongoing weight loss was dramatic (BMI was as low as 17.5). When we noticed a stabilization of the body weight under ruxolitinib and lacking other validated biomarkers in this rare disease, we chose to include body weight as a possible indication for clinical stabilization (BMI eventually went up to 21.5).

In conclusion, we provide promising evidence that JAK inhibition could qualify as a treatment option to slow clinical worsening in symptomatic RVCLS adults. *CXCL10* transcripts in PBMCs and ANAs levels could serve as valuable biomarkers to monitor treatment attempts.

## Data availability statement

The original contributions presented in the study are included in the article/Supplementary material, further inquiries can be directed to the corresponding author.

## Ethics statement

The studies involving human participants were reviewed and approved by Ärztekammer Hamburg (PV4405). The patients/participants provided their written informed consent to participate in this study. Written informed consent was obtained from the individual(s) for the publication of any potentially identifiable images or data included in this article.

## Author contributions

FU and MF conceived the study, collected and analyzed data, took care of the patients, and wrote the manuscript. FU and SZ performed the experiments. MA reviewed and commented on immunological assays. MF supervised and funded the study. All authors critically reviewed the manuscript and agreed to its submission.
