# Supplementary material for: Case report: JAK inhibition as promising treatment option of fatal RVCLS due to TREX1 mutation (pVAL235Glyfs*6)
Source: Front Neurol. 2023 Feb 21;14:1118369. doi: 10.3389/fneur.2023.1118369 (PMC9989011; doi:10.3389/fneur.2023.1118369)
Supplement: Supplementary file 1 [file Data_Sheet_1.pdf]

Supplementary Material

Case report: JAK inhibition as promising treatment option of fatal RVCLS due to *TREX1* mutation (pVAL235Glyfs*6)

Friederike Ufer[1], Susanne M. Ziegler[2], Marcus Altfeld[2], Manuel A. Friese[1*]

[1]Institute of Neuroimmunology and Multiple Sclerosis, University Medical Center Hamburg-Eppendorf, Hamburg, Germany

[2]Department of Virus Immunology, Leibniz Institute for Virology, Hamburg, Germany.

[*]Correspondence: manuel.friese@zmnh.uni-hamburg.de

1 Supplementary Data

Case synopsis of index patient

Clinical history

In February 2011 the female index patient had started to feel exhausted and experienced a general muscle weakness, severe headaches, hair loss, amenorrhea, arthralgias of the hip and knees and visual abnormalities that started at the age of 41. After admission to the ophthalmological department, she was diagnosed with retinal vasculitis in 2012 and prednisolone as immunosuppressant therapy was administered. In 2013 two episodes occurred with weeklong weakness and numbness of the right arm. A first neurological admission did not reveal any abnormalities. With elevated ANA titers a systemic vasculitis was suspected and several immunosuppressive therapies (see below) were tried. They all failed to stop progression of the symptoms and the patient experienced a progressive loss of visual acuity in combination with massive oedema at the lower legs. In order to treat her progressive loss of vision, both eyes were treated with repetitive trabeculectomy, laser coagulation, intraocular avastin injections and intraocular healon-injections.

Neurological examination at first presentation (2014) showed reduced visual acuity (left eye: < 0.05 right eye: 0.4), a restriction of the visual fields to the right, gait abnormalities without paresis of the upper or lower extremities.

Immunosuppressant treatment history:

05/2012 – 08/2012	Prednisolone (40 mg per day)
07/2012 – 09/2013	Azathioprine (100 mg per day)
08/2012 – 09/2013	Cyclosporine A
09/2013 – 12/2013	Cyclophosphamide (6 cycles with each 630 mg)
02/2014 – 03/2014	Methotrexate (15 mg per week)
02/2014 – 03/2014	Azathioprine (50–100 mg per day)
04/2014 – 09/2014	Prednisolone (2,5–30 mg per day)
02/2015 – 01/2016	Emtricitabine (200 mg per day),<br>Tenofovir disoproxil fumarate (245 mg per day),<br>Nevirapine (400 mg per day)
Since 03/2016	Ruxolitinib (10–25 mg per day)

1.1 Supplementary Figures and Tables

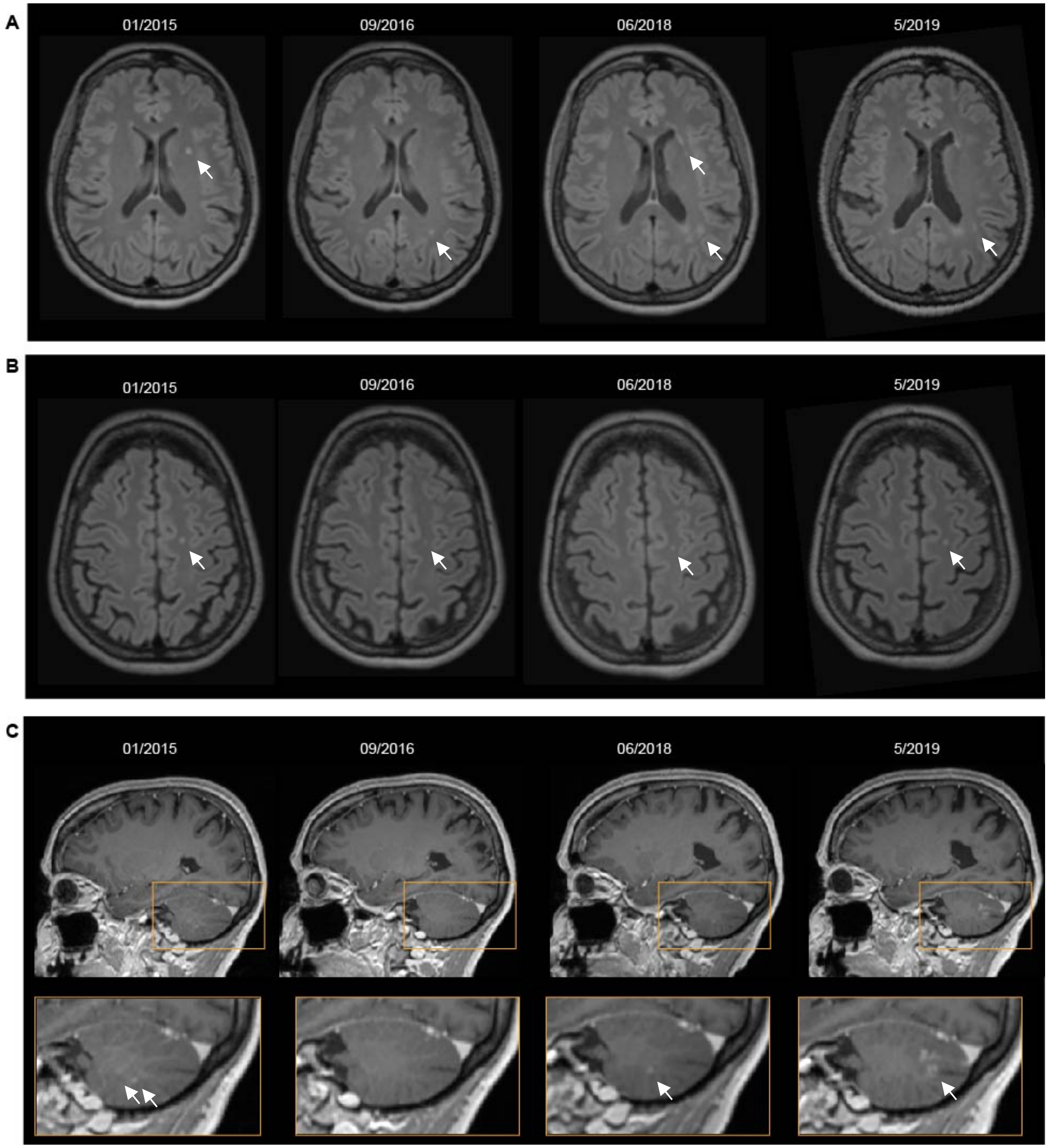

Supplementary Figure 1: Cerebral MRI images of the index patient over time. (A and B) T2/FLAIR-weighted images showing punctate lesions at indicated time points. (C) Gadolinium-enhanced T1-weighted images with magnification of the cerebellum (bottom) at indicated time points. Prominent lesions are indicated with an arrow.

Supplementary Table S1: Characterization of family members

**Demographic characteristics**		
Number of studied family members[1]	29	
Sex of reported family members	69% female (20/29)	31% male (9/29)
Stillbirth with unknown sex	6	
Symptomatic individuals	59% (17/29)	
Sex of symptomatic individuals	76% female (13/17)	24% male (4/17)
Number of genetically confirmed mutation carriers	41% (12/29)	
Symptomatic mutation carriers	75% (9/12)	
Asymptomatic mutation carriers (current age in year)	25% (3/12) (46, 28 and 26)	
Genetically confirmed non-mutation carriers	20% (6/29)	
Living symptomatic individuals	47% (8/17)	
Deceased symptomatic individuals	52% (9/17)	
Age of deceased symptomatic individuals (mean ± SD)	52 ± 9.8 years	
Age at time of death in symptomatic individuals in generation I +II (mean ± SD)	61.1 ± 2.3 years	
Age of deceased symptomatic individuals in generation III (mean ± SD)	42 ± 2.8 years	
**Clinical characteristics[1] of symptomatic individuals**		
Cerebral abnormalitiy[2]	58% (10/17)	
− Isolated cerebral abnormality	29% (5/17)	
Retinopathy	58% (10/17)	
− Isolated retinopathy	35% (6/17)	
Kidney disease[3]	17% (3/17)	
− Isolated kidney disease	6% (1/17)	
Combination of cerebral abnormality and retinopathy	23% (4/17)	
Combination of cerebral abnormality and kidney disease	6% (1/17)	
Combination of cerebral kidney disease and retinopathy	12% (2/17)	
Combination of cerebral abnormality, kidney disease and retinopathy	6% (1/17)	

Unless indicated otherwise, the first number is shown as percentage of subjects followed in brackets by the number of affected subjects and the in each case relevant denominator that varies according to the number at risk for the specific question. Mutation carriers show pVAL235Glyfs*6 of *TREX1*.

[1]Either clinical and diagnostic examination or gathered by medical records with or without personal telephone call or telephone call to treating physicians

[2]Progressive focal neurological symptoms, cognitive impairment or psychiatric disease

[3]Subject to dialysis

Supplementary Table S2: Clinical characterization of index patient

**Demographic characteristics**	
Current age (years)	53
Sex	female
Age at clinical disease onset (years)	41
Age at diagnosis of RVCLS (years)	45
Age at experimental therapy start (years)	46
**Clinical characteristics of RVCLS**	
Age at eye affection	41
Age at cerebral manifestation	45
Kidney disease	Not evident until 08/2021
**Other relevant diagnoses/symptoms**	
Coxarthrosis right	
Osteopenia	
Latent tuberculosis	
Asthma bronchial	
Allergic rhinitis	
Lymphopenia	
Macrocytic anemia	
Migraine	

RVCLS = Retinal vasculopathy with cerebral leukoencephalopathy and systemic manifestations

Supplementary Table S3: Key laboratory findings of index patient

Laboratory parameters (unit)	Reference	22.07. 2020	25.01. 2019	21.09. 2018	08.06. 2017	22.12. 2016	24.06. 2016	22.01. 2016	13.11. 2016	23.01. 2015
**Hematology**										
**Hemoglobin (g/dL)**	12.3–15.3	**11.1 –**	**11.5 –**	**11.4 -**	**10.4 –**	**10.1 –**	**10.2 –**	12.5	13.0	12.6
**Hematocrit (%)**	35–45	35.0	**34.0 –**	35.8	**30.2 –**	**30.6 –**	**30.4 –**	38.3	41.5	38.5
**Erythrocytes (bn/L)**	4.10–5.10	**3.34 –**	**3.34 –**	**3.50 –**	**3.03 +**	**3.00 –**	**2.98 –**	**3.60 –**	**3.79 –**	**3.83 –**
**MCV (fL)**	80.0–94.0	**105 +**	**102 +**	**102 +**	**99.5 +**	**102.1 +**	**102.1 +**	**106.3 +**	**109.4 +**	**100.5 +**
**MCH (pg)**	26.0–34.0	33.2	**34.4 +**	32.6	**34.3 +**	33.7	**34.2 +**	**34.7 +**	**34.3 +**	32.9
**MCHC (g/dL)**	31.5–37.0	31.7	33.9	31.8	34.5	33.0	33.5	32.7	**31.4 –**	32.7
**EVB (%)**	11.5–14.5	13.8	13.6	14.4	**14.8 +**	14.2	14.1	13.4	13.6	13.6
**Leucocytes (bn/L)**	3.8–11.0	**2.6 –**	4.0	4.6	**3.4 –**	**3.7 –**	**3.4 –**	4.1	**3.7 –**	5.1
**Thrombocytes (bn/L)**	150–400	200	173	233	247	260	235	172	172	172
**Neutrophiles (bn/L)**	1.50–7.70		2.80	3.13		2.13	2.00	2.70	2.01	3.13
**Lymphocytes (bn/L)**	1.1–3.4		**0.81 –**	**0.94 –**		1.10	**0.95 –**	**1.01 –**	**1.08**	1.14
**Monocytes (bn/L)**	0.2–0.9		**0.19 –**	0.29		**0.19 –**	0.28	**0.18 –**	0.23	0.22
**Eosinophiles (bn/L)**	0.1–0.5		0.10	**<0.10 –**		0.16	**<0.10 –**	0.19	**0.33**	0.48
**Basophiles (bn/L)**	–0.10		<0.10	<0.10		<0.10	<0.10	<0.10	<0.10	<0.10
**Neutrophiles (%)**			70.4	68.2		57.5	58.5	65.6	54.0	61.3
**Lymphocytes (%)**		20	20.3	20.5		29.7	27.8	24.5	29.0	22.4
**Monocytes (%)**		8	4.7	6.2		5.1	8.2	4.4	6.2	4.4
**Eosinophiles (%)**		3	2.6	1.7		4.2	2.2	4.5	9.0	9.3
**Basophiles (%)**		1	0.2	0.3		0.2	0.3	0.4	0.4	0.3
**Rod neutrophiles (%)**		1								
**Plasma/serum**										
**Potassium (mmol/L)**	3.5–5.0				4.3					3.8
**Calcium (mmol/L)**	2.13–2.63				2.22					2.24
**Inorg. phosphor (mmol/L)**	0.7–1.50				**0.71 –**					
**Albumin (g/dL)**	35–50				36.3					**34 –**
**Urea (mg/dL)**	7–19				13	17			12	
**Creatinine (mg/dL)**	0.5–1.0			0.93	0.82	0.82		0.75	0.65	
**Uric acid (mg/dL)**	2.5–6.0					3.7		3.5	3.0	
**Cholesterol (mg/dL)**	150–200			**267 +**						
**Triglyceride (mg/dL)**	70–180			138						
**HDL-Cholesterol (mg/dL)**	45–65			**90 +**						
**LDL-Cholesterol (mg/dL)**	–150			149						
**AST (U/L)**	10–35			24		23	30	22	23	17
**ALT (U/L)**	10–35			28		23	32	19	35	15
**GGT (U/L)**	–38			25		17	27	34	**43 +**	12
**CRP (mg/L)**	–5					<5			<5	<5
**ANA - titer**	<1:80	**1:160**		**1:320**					**1:1280**	**1:5120**
**Vitamin B12 (ng/L)**	197–866						862			
**Urine**										
**Albumin/urine (mg/L)**				12.0	15.4					
**Protein (mg/dl)**	<119			114.8	83.4					
**Creatinine (g/L)**				0.87	0.94					
**Specific weight (g/mL)**	1.002–1.040			1.016	1.011					1.012
**pH**	4.5-7.5			6.5	5.5					6.0
**Glucose**	Negative			neg	neg					neg
**Ketone**	Negative			neg	neg					neg
**Leucocytes**	Negative			neg	neg					neg
**Nitrite**	Negative			neg	neg					neg
**Protein**	Negative			neg	neg					neg
**Hemoglobin**	Negative			neg	neg					neg
**Urobilinogen**	Normal			normal	normal					neg
**Bilirubin**	Negative			neg	neg					neg
**GFR (mL/min)**				72	78.9	79.1		>60	>60	

**Cerebrospinal fluid**										
**Cell count**	1-4									2
**Glucose (mg/L)**	320–820									540
**Protein (mg/L)**	140–500									357
**Lactate (mmol/L)**	1.5–2.1									2.0
**Intrathecal IgG index**	< 0.01%									< 0.01%
**Intrathecal IgA index**	< 0.01%									< 0.01%
**Intrathecal IgM index**	< 0.01%									< 0.01%
**Oligoclonal bands**	Negative									neg

neg = negative

Abnormal findings are depicted in bold with “+” marking values above reference and “–“ below reference.
